# Risk factors for bovine Tuberculosis at the national level in Great Britain

**DOI:** 10.1186/1746-6148-8-51

**Published:** 2012-05-07

**Authors:** Paul R Bessell, Richard Orton, Piran C L White, Mike R Hutchings, Rowland R Kao

**Affiliations:** 1 Institute of Biodiversity, Animal Health and Comparative Medicine, College of Medical, Veterinary and Life Sciences University of Glasgow, 464 Bearsden Rd, Glasgow, G61 1QH, UK; 2 Boyd Orr Centre for Ecology and Ecosystem health, College of Medical, Veterinary and Life Sciences University of Glasgow, 464 Bearsden Rd, Glasgow, G61 1QH, UK; 3 The Roslin Institute, The University of Edinburgh, Easter Bush, Midlothian, EH25 9RG, UK; 4 Environment Department, University of York, Heslington, York, YO10 5DD, UK; 5 SAC, Roslin Institute Building, Easter Bush, Midlothian, EH25 9RG, UK

## Abstract

**Background:**

The continuing expansion of high incidence areas of bovine Tuberculosis (bTB) in Great Britain (GB) raises a number of questions concerning the determinants of infection at the herd level that are driving spread of the disease. Here, we develop risk factor models to quantify the importance of herd sizes, cattle imports from Ireland, history of bTB, badgers and cattle restocking in determining bTB incidence. We compare the significance of these different risk factors in high and low incidence areas (as determined by parish testing intervals).

**Results:**

Large herds and fattening herds are more likely to breakdown in all areas. In areas with lower perceived risk (longer testing intervals), the risk of breaking down is largely determined by the number of animals that a herd buys in from high incidence areas. In contrast, in higher perceived risk areas (shorter testing intervals), the risk of breakdown is defined by the history of disease and the probability of badger occurrence. Despite differences in the management of bTB across different countries of GB (England, Wales and Scotland), we found no significant differences in bTB risk at the national level after these other factors had been taken into account.

**Conclusions:**

This paper demonstrates that different types of farm are at risk of breakdown and that the most important risk factors vary according to bTB incidence in an area. The results suggest that significant gains in bTB control could be made by targeting herds in low incidence areas that import the greatest number of cattle from high incidence areas.

## Background

The control of an infectious disease is often complicated when more than one host species are involved and one of those is a wildlife reservoir. This problem is exacerbated if the reservoir host is poorly understood or if the disease is difficult to manage in the reservoir population [1,2]. One of the best described examples of this is that of bovine Tuberculosis (bTB) in Great Britain (GB), caused by *Mycobacterium bovis*. Largely eliminated in many industrialised countries, in GB the disease was eliminated from most cattle herds during the 1960s [3]. However, since then prevalence has been gradually rising and the area affected growing in size [4]. It was soon suspected that this rise was related to the presence of the Eurasian badger (*Meles meles*), once it was recognised that badgers were a host for *M. bovis*[5,6]. However, the nature of the relationship to cattle bTB remained controversial and so the randomised badger culling trial (RBCT) was set up to investigate the effectiveness of badger culls as a control for bTB spread [7]. While the RBCT showed that badger culling operations and cattle herd breakdowns rates are strongly and significantly associated with each other [8-10], relatively little is understood about the role of badgers in herd breakdowns across GB, though a recent paper has analysed risk factors for badger presence [11]. These analyses have been extrapolated to produce a prediction of badger distribution in GB, features that have previously been suggested could be used to predict risk of bTB to cattle [12].

Bovine TB cases are identified using the Single Intradermal Comparative Cervical Test (SICCT) or “skin test” which has relatively low sensitivity [13]. An animal reacting positively to the skin test is called a reactor and is slaughtered. Further, limited *ante-mortem* testing is carried out using gamma-interferon test, which is believed to have greater sensitivity [14]. Furthermore, carcasses of slaughtered animals are inspected at the abattoir for lesions that could indicate bTB. The case is confirmed by successfully culturing the pathogen from a suspect lesion *post-mortem.* Routine testing is carried out using the skin test on a one-to-four yearly basis depending upon local disease incidence. Additional testing is carried out following a herd breakdown, on neighbouring herds and on herds with traced contacts with breakdown herds. Cattle moved from high risk areas are also tested before movement to other areas; further, all cattle from England and Wales are tested before and after movement to Scotland.

The number of detected bTB breakdowns in GB has been rising year-on-year for a number of years, punctuated by a decrease in cases in 2001 caused by the epidemic of foot-and-mouth disease (FMD) in GB that restricted the number of tests that could be conducted that year [15]. The culmination of the year-on-year rise in incidence was in 2008 (the peak in incidence), when 2,764 herd breakdowns were confirmed (a prevalence of 5%) with a total of 39,302 animals slaughtered as reactors, and with movement restrictions imposed upon 7,957 (9.2%) herds [4]. Although the pattern of increase and spread in bTB is well documented, the reasons for this increase remain unclear.

While badgers are now recognised as a critical component for bTB persistence, the contribution of badgers to increasing incidences of bTB has not been quantified. The restocking of herds following the 2001 FMD epidemic has been demonstrated to have facilitated the spread of bTB to new areas [16]. However, restocking caused by FMD only explains a small part of the bTB expansion and various studies have looked into risk factors for herd breakdowns to assist understanding of the expansions of these hotspots [15,17-20]. There are a number of potential routes of transmission and previous studies [15,18] have demonstrated that cattle movements are important for transmission, with an estimated 15% of herd breakdowns are due to recorded movements of cattle, 75% of breakdowns to local effects within High Risk areas (HRAs), and 9% unexplained [18]. These differences will vary with the local disease prevalence, with movements and the unexplained component being most important in three and four year testing areas. In addition to movements of potentially exposed animals within GB, there are a large number of imports of cattle from Ireland. Despite post-import testing of animals arriving from Ireland, this is likely to represent a risk of introduction of disease from outside GB due to the arrival of falsely negative animals [17].

Specific risk factor studies have identified larger herds as both a risk factor for having a breakdown [20-22] and for the duration of the breakdown [23]. Movements of animals to and from high incidence areas [16,24], and from farm sales [19,21] have also been demonstrated to be associated with breakdowns. Other factors associated with herd breakdowns include being a dairy farm and various farming practices such as operating over multiple premises, the spreading of fertilizers and the area of land farmed [7,16,19,20,25]. However, all of these analyses have been applied to small portions of high incidence areas. Thus far, no studies have looked at risk factors at the national level and in particular none have compared them to those in lower incidence areas.

**Figure 1:**
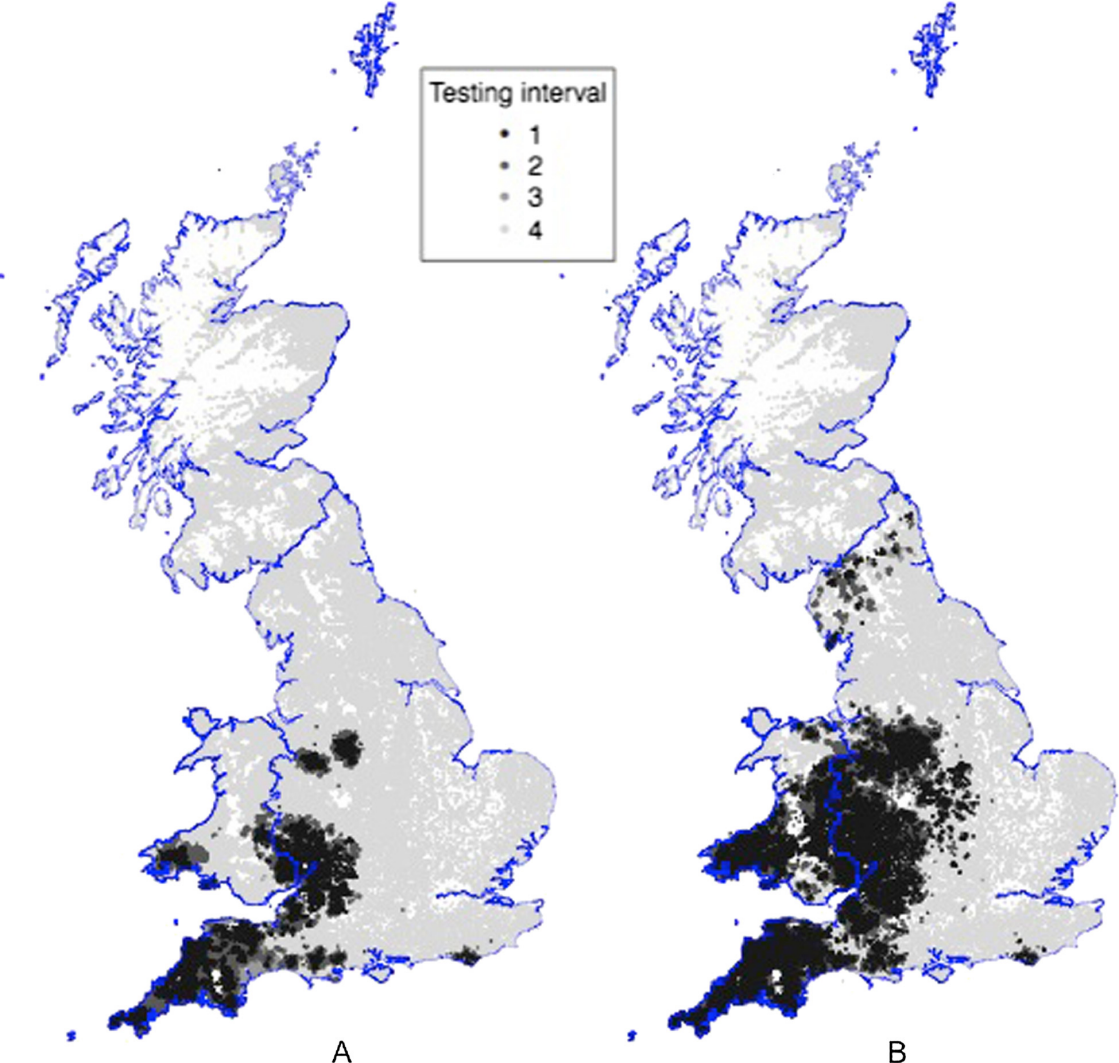
The distribution of farms by parish testing interval in 2002 (A) and 2008 (B).

The extent of the bTB endemic area in GB continues to expand as identified by changes in the parish testing intervals (Figure 1). Under the bTB control policy in operation prior to 2009, all herds are routinely tested on a one, two, three or four year routine herd testing (RHT) interval based upon the perceived risk in the parish of the farm, or the risk in neighbouring parishes. The testing period of the parish is reviewed on a quarterly basis and the extent of the one and two yearly tested parishes are considered high risk areas (HRAs), as they are intended to reflect the current extent of bTB endemicity.

Based upon this, the aims of this paper are:

1. To establish the role of imports of cattle from Ireland in the transmission of bTB.

2. To establish whether an estimate of badger distribution can serve as a predictor of bTB incidences.

3. To investigate whether herds that had animals culled during the 2001 FMD epidemic and were subsequently restocked are more likely to have a bTB breakdown.

4. To identify whether different determinants of infection operate in high incidence areas compared to low incidence areas.

5. To investigate whether differences in risk of infection remain between England, Wales and Scotland after these risk factors have been taken into account.

## Results

**Figure 2:**
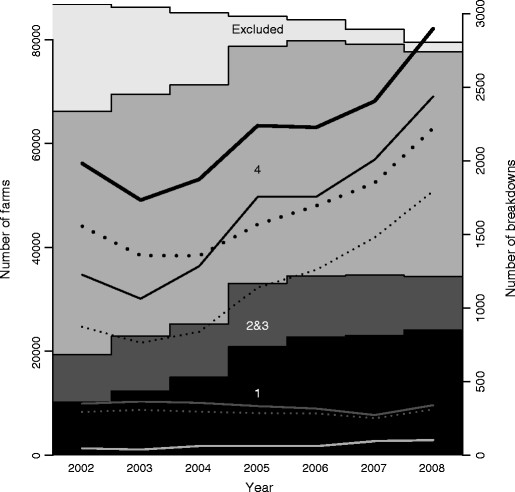
**The total number of active herds (solid areas) and the total number of breakdowns (lines) in each year between 2002 and 2008, the broken lines correspond to the number of “new” breakdowns – the breakdowns that are included in these analyses.** The dark grey area and lines correspond to areas that were in one year testing, above the dark grey area and corresponding lines those that were in 2 and 3 year testing areas, above those holdings that were in four year testing between 2002 and 2008. The top block and lightest grey lines are those that were excluded at that time due to being in four year testing but subsequently removed from the analysis for that particular year.

**Figure 3:**
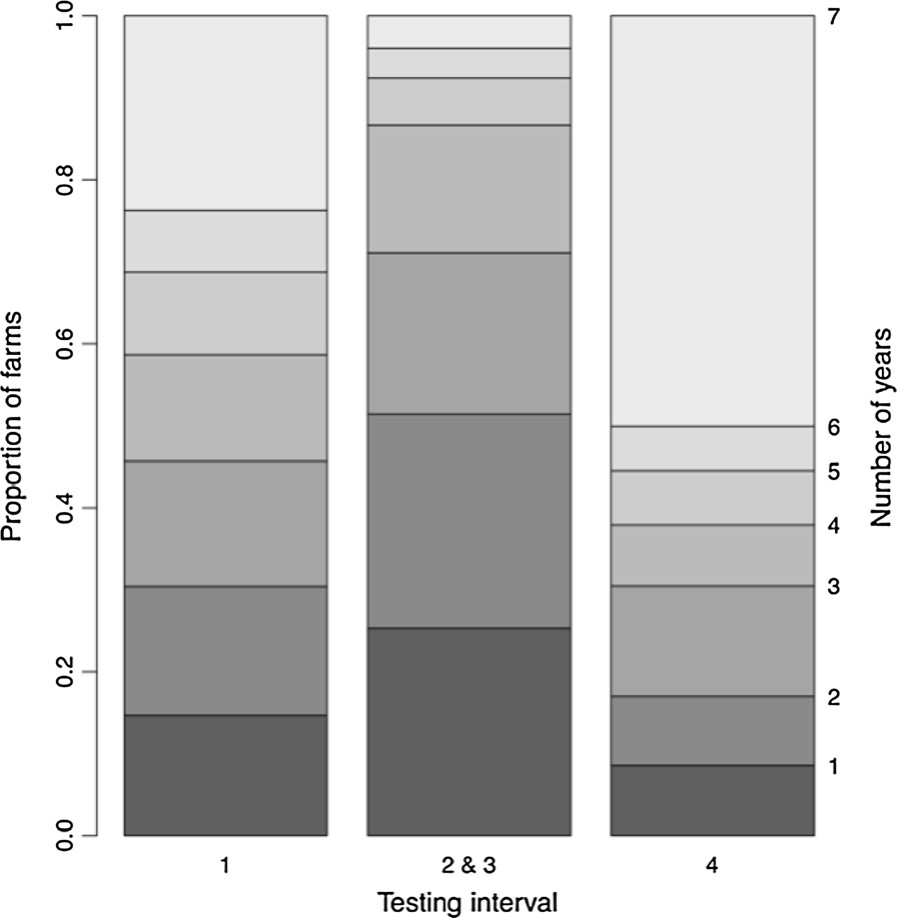
**Stacked barplot of the number of years that each holding spends in the three interval categories.** The numbers on the right hand side are the numbers of years spent in each testing interval. The y-axis (left hand) is the proportion of all herds in the column.

A total of 15,358 bTB breakdowns were confirmed between 2002 and 2008 inclusive, of which 11,599 were not recorded as part of a follow-up and so can be considered a new breakdown for the purposes of these analyses (Table 1). The number of herds in GB is declining, whilst the number of breakdowns is increasing (Figure 2). Furthermore, the number of herds in 1 year testing parishes (high incidence parishes) is increasing at the expense of those in 4 year testing parishes (Figure 2). Analysis of the period of time spent in each parish testing interval shows that the majority of herds in 4 year testing intervals are there for the duration of the study period (Figure 3). However, intervals 2 and 3 are to a much greater extent a transitional testing interval, with the majority of herds spending only one or two years in 2 and 3 year testing intervals (Figure 3).

**Table 1:** The distribution of the categorical variables from these analyses, all numbers are farm years

		Testing interval							
	**Unit**	**All herds**		**1 year**		**2,3 year**		**4 year**	
		**Non-TB**	**TB**	**Non-TB**	**TB**	**Non-TB**	**TB**	**Non-TB**	**TB**
Country	England	373849	9068	91768	6607	52630	1491	187359	284
	Wales	102840	2400	25107	1539	20908	539	95854	131
	Scotland	95854	131	0	0	0	0	34143	52
Herd type	Beef	213231	3566	44569	2649	24199	552	124649	132
	Fatt’	58847	1163	10286	764	6133	181	37872	115
	Suckler	167501	2770	31721	1927	22144	515	92457	102
	Dairy	113234	3807	26576	2618	18435	728	51255	96
	Store	19730	293	3723	188	2627	54	11123	22
Herd size	0 – 10	137438	427	27406	320	17158	61	78065	18
	11 – 100	242294	4021	51514	2984	31853	660	130805	90
	>100	192811	7151	37955	4842	24527	1309	108486	359
Irish imports	0	567291	11505	116560	8116	73312	2019	312810	423
	>0	5252	94	315	30	226	11	4546	44
History	No	556467	9744	103245	6430	72053	1923	317053	464
	Yes	16076	1855	13630	1716	1485	107	303	3
High risk moves	0	290232	393	15031	164	9958	39	246715	147
	1-10	102995	923	22602	622	14758	122	45405	62
	>10	179316	10283	79242	7360	48822	1869	25236	258
FMD	No	561173	11296	114834	7958	72219	1970	310725	448
	Yes	11370	303	2041	188	1319	60	6631	19
Badgers	Mean prob'	0.259	0.281	0.293	0.295	0.283	0.286	0.223	0.219
**Block Totals**		**572543**	**11599**	**116875**	**8146**	**73538**	**2030**	**317356**	**467**

The models created for each of the four analyses show different compositions and all have good fits as indicated by the area under the receiver operator characteristic (ROC; Table 2). The residuals of the models were checked for spatial dependency and none was found. In all four models, fattening herds were significantly different from all of the other herd types. In the whole of GB model and the one year testing interval model there was a significant interaction (a protective effect) between the x coordinate and the y coordinate which means that risk of infection decreases with distance from the south-west corner of GB. Herd size and bringing animals in from high risk areas were risk factors in all four models, whilst bringing in animals from Ireland was a risk factor in four year testing areas, but not in other testing areas due, in part to the low numbers moving into 1, 2 and 3 year testing areas (Table 3).

**Table 2:** Multivariable mixed logistic regression models for infection with bTB between 2002 and 2008 fitted for the four different models. “-“represents variables that were not significant so not included in that model, “REF” indicates (part of) the reference level., “OR” in the odds ratio and numbers in brackets are the 95% confidence interval

		Testing interval							
	**Unit**	**All herds**		**1 year**		**2,3 year**		**4 year**	
		**OR**	**p**	**OR**	**p**	**OR**	**P**	**OR**	**p**
Country	England	1	REF	1	REF	-	-	1	REF
	Wales	0.919 (0.86, 0.98)	0.008	0.889 (0.74, 0.89)	<0.001	-	-	1.352 (0.99, 1.84)	0.055
	Scotland	0.822 (0.64, 1.05)	0.119	-	-	-	-	3.709 (2.59, 5.31)	<0.001
X coord	x10^-5^	1.279 (1.22, 1.34)	<0.001	1.377 (1.28, 1.49)	<0.001	-	-	-	-
Y coord	y10^-5^	1.123 (1.05, 1.21)	<0.001	1.179 (1.03, 1.35)	0.016	0.856 (0.83, 0.90)	<0.001	1.125 (0.92, 1.37)	0.241
Testing interval	1	1	REF	-	-	-	-	-	-
	2	0.445 (0.42, 0.47)	<0.001	-	-	1	REF	-	-
	3	0.212 (0.16, 0.27)	<0.001	-	-	0.455 (0.35, 0.59)	<0.001	-	-
	4	0.181 (0.17, 0.19)	<0.001	-	-	-	-	-	-
Herd type	Beef	1	REF	1	REF	1	REF	1	REF
	Fatt’	1.221 (1.14, 1.31)	<0.001	1.254 (1.15, 1.37)	<0.001	1.214 (1.03, 1.43)	0.023	1.942 (1.54, 2.45)	<0.001
	Suckler	1	REF	1	REF	1	REF	1	REF
	Dairy	1	REF	1	REF	1	REF	1	REF
	Store	1	REF	1	REF	1	REF	1	REF
Herd size	0 – 10	1	REF	1	REF	1	REF	1	REF
	11 – 100	2.059 (1.84, 2.31)	<0.001	2.976 (2.56, 3.46)	<0.001	3.366 (2.43, 4.66)	<0.001	2.306 (1.35, 3.95)	0.002
	>100	4.026 (3.59, 4.52)	<0.001	5.712 (4.87, 6.71)	<0.001	7.616 (5.44, 10.6)	<0.001	7.654 (4.57, 12.8)	<0.001
Irish imports	0	1	REF	-	-	-	-	1	REF
	>0	1.760 (1.41, 2.19)	<0.001	-	-	-	-	4.005 (2.85, 5.63)	<0.001
Badgers	Count/10	1.060 (1.04, 1.09)	<0.001	1.070 (1.03, 1.11)	<0.001	-	-	-	-
History	No	1	REF	1	REF	1	REF	-	-
	Yes	1.379 (1.30, 1.46)	<0.001	1.369 (1.28, 1.46)	<0.001	1.750 (1.41, 2.17)	<0.001	-	-
High risk moves	0	1	REF	1	REF	1	REF	1	REF
	1-10	3.342 (2.95, 3.79)	<0.001	1.410 (1.16, 1.71)	<0.001	1.230 (0.83, 1.83)	0.306	1.678 (1.24, 2.27)	<0.001
	>10	7.463 (6.61, 8.43)	<0.001	2.020 (1.66, 2.47)	<0.001	2.261 (1.52, 3.36)	<0.001	8.726 (6.85, 11.1)	<0.001
FMD	No	1	REF	-	-	1	REF	-	-
	Yes	1.217 (1.07, 1.38)	0.002	-	-	1.469 (1.11, 1.94)	0.007	-	-
x*y		0.917 (0.90, 0.94)	<0.001	0.903 (0.87, 0.94)	<0.001	-	-	-	-
**Area under the ROC**		**0.896**		**0.767**		**0.792**		**0.912**	

**Table 3:** Movements of animals from Ireland onto herds during the previous year broken down by parish testing interval. Note the units are herd-years - herds are replicated across all 7 years between 2002 and 2008

		Testing interval		
		1 (%)	2&3 (%)	4 (%)
Years with Irish Imports	0	128,044	75,634	378,860
	>0	359 (0.3)	239 (0.3)	4,765 (1.2)

The size of the local cattle population was a significant risk factor as a univariate predictor, however once incorporated in the model along with the herd size category, the sign changes and the predictor becomes a significant protective effect. This is due to a correlation between the number of cattle in the 100 km^2^ cells and the herd size covariate, due to a clustering of larger herds. There was an additional correlation between badger numbers and the local cattle density (R^2^ = 0.244, p < 0.001). As the number of animals on a herd usually changes during the year, sensitivity analysis compared animal numbers on 1^st^ January with those on 1^st^ June and showed no significant change to risk factor estimates. The estimated probability of badger occurrence was a significant risk factor in the highest bTB incidence areas (1 year testing) and the history of bTB was a significant risk factor in 1, 2 and 3 year testing areas. Neither of these factors were significant in the lowest bTB incidence (four year testing) areas. Sensitivity analysis compared the implementation of history of infection here – infections between 1997 and 2001 versus a history of infections in the previous two years. There was no significant change in the odds ratios between the two implementations. Having had animals culled during the 2001 FMD epidemic was only significant in two and three year testing areas.

## Discussion

As the incidence of bTB continues to rise in GB it is important to develop an improved understanding of the characteristics of the herds that are becoming infected. This can aid control and surveillance of the disease to help to ensure that the disease is contained with minimum surveillance effort. The analyses presented in this paper have demonstrated that it is possible to identify herds that are likely to have a bTB breakdown. Variables associated with the risk of infection appeared to vary depending upon the local incidence of disease. In high incidence parishes (the one year testing parishes) it is features of the locality (badger density and the history of bTB) that define the likelihood of infection. In low-incidence areas (the four year testing parishes) the risk of infection is defined solely by herd demographics and whether the herd brings in high-risk animals from other areas of GB or from Ireland; animals from Ireland predominantly arrive in four year testing parishes (Table 2). The significance of these high risk imports underlines the need to a more sensitive test for bTB as these animals are tested prior to movement.

Whilst demographic factors are important in one year testing parishes, the risk of recording a breakdown is also defined by the probability of badger presence and whether there is a history of bTB on the herd between 1997 and 2001 – the period prior to the analysis in this study. This is the first time that badger-related factors have been shown to be important at a national scale. A sensitivity analysis that defined a history of infection as the two years prior to year *k* did not result is a significant change from analysis of the period 1997–2001. This indicates that the at-risk farms have been defined prior to the period of study and that the risk of infection is not dynamic but static. This opens up attractive options for risk-based surveillance of the at risk holdings by targeting such herds. In medium incidence (two and three year testing parishes) badgers are not significant as a risk factor, but a history of infection is. Additionally herds that had animals culled during the 2001 FMD epidemic are a significant risk factor in these areas, but not in others, which may be a result of where herds sourced their stock following the FMD epidemic. It may also be a result of disease being seeded in areas that had the properties to sustain infection over a prolonged period, or the effect being overwhelmed by an increase in incidence.

Only fattening herds were significantly different from the other herd types. There are several reasons for this. As fattening herds typically source only older stock and source them from a variety of sources they are likely to act as “sinks” for infection. Furthermore, as fattening herds send relatively large numbers of stock to the slaughterhouse, infection is more likely to be detected on fattening herds via abattoir surveillance and indeed, a greater proportion of breakdowns on fattening herds are identified at the slaughterhouse. Therefore the amount of testing is heterogeneous compounded by many fattening herds being exempt from routine herd testing due to their high stock turnover rate. However, for this reason they are also likely to be less important epidemiologically; the animals go to slaughter and as there are likely to be fewer movements off the fattening herds onto other herds, so fattening herds form the end of the transmission chain. The significance of fattening herds is in contrast with the results of Vial et al. [20] who found greater risk of breakdowns among dairy herds as well as larger herds. However, the study of Vial et al. [20] was restricted to much smaller areas and did not include movements of animals. This may explain the differences between the sets of results.

These results support the work of Green et al. [18] who demonstrated that 75% of cases are due to local effects (the additional local effects, such as badgers and the history of bTB, seen in high, and to a lesser extent, medium bTB incidence areas), whilst 15% are due to recorded cattle movements (dominating the risks seen in lower bTB incidence areas). The remaining 9% of unexplained breakdowns may be the result of heterogeneities in herd risk, reflecting the demographic factors seen here, in particular herd size and fattening herds and their potentially older age distribution.

Despite the disease being managed independently by the respective governments in England, Scotland and Wales, few differences were found between the three countries in terms of relative risk of infection. However, when just the four year-testing regions of GB are compared, there is a significantly greater risk of breakdown in Scotland relative to England and Wales. This is likely to be due to the significant protective effect of the y-coordinate in the model. There are large numbers of non-breakdown herds in four year testing areas of northern England, however, there have been a large cluster of breakdown herds in four year testing areas of southern Scotland, the significant difference of Scotland is therefore offsetting the effect of the y-coordinate protective effect in these areas. If the y-coordinate is dropped from the model, Scotland is no longer significant. Generally, despite the different prevalences and management strategies, there are no national level differences in bTB risks.

A key consideration when considering the role of wildlife reservoirs is whether or not the ‘spillover host’ (in this case, the cattle population) can sustain the disease epidemic on their own or produce transmission chains resulting in locally important problems. While it is known that transmission chains can occur [17], here we show that cattle alone are unlikely to sustain an epidemic under the existing test and slaughter regime; there is only a distinct and relatively stable subset of herds that can be defined as being at elevated risk of breaking down. In areas perceived to be at low risk, these factors are largely demographic rather than epidemiological. Indeed, whilst the factors that define the risk of infection vary depending upon the local prevalence of disease, the factors do not vary depending upon the country of GB. This shows that management factors are of secondary importance and that environmental factors are defining transmission and spread, though this analysis does not preclude within-herd transmission factors being associated with identified risks such as herd history. Therefore, control may be enhanced and streamlined by developing a risk-based strategy for bTB surveillance. In low risk areas the disease can be controlled by monitoring “risky herds” and potential high risk movements of animals and imports. However, this study has also demonstrated that there are no differences between the at-risk herd types in high risk areas compared with low risk areas; it is merely the environmental conditions that determine the risk in those areas. Therefore, should the specific environmental conditions become established in low risk areas then bTB may become established in these areas.

## Conclusion

This paper has demonstrated that it is possible to identify cattle herds that are more likely to have a bTB breakdown, but the determinants of breakdown change as the background risk of infection changes. Within perceived higher risk areas it is principally locality factors that determine the risk of breakdown, whilst in lower risk areas the principal determinants are the amount of contacts that herds have with higher risk herds and animals. These determinants of infection could be used to develop a strategy for risk-based surveillance for bTB.

## Methods

### Data

For the purposes of these analyses the term “holding” was used to describe the physical attributes of a farm enterprise – identified by a unique County-Parish-Holding (CPH) number. The term “herd” was used to describe the group of cattle that are housed on a holding at any given time. Data on holdings and disease were taken from the 2009 extract of the Defra animal health information system (VetNet). The herd table from VetNet includes data of herd type, easting and northing coordinates which typically represent the main farm buildings of the holding and the years for which the holding was active. A dataset comprising each herd that was recorded as being active in the VetNet herd table in each year between 2002 and 2008 (inclusive) was constructed. The unique identifier for each herd and holding was the CPH number and it was ensured that each CPH number was unique in each year. The VetNet incidents table was used to identify each herd that recorded a new confirmed breakdown during each year between 2002 and 2008.

The aim of the paper is to identify factors associated with new confirmed breakdowns, so breakdowns that were identified by follow-up testing following an earlier breakdown were not included. These follow-up tests included those at 3, 6 and 12 months following resolution of a bTB breakdown.

Data on movements of animals to each holding are recorded on the British Cattle Movement System (BCMS) Cattle Tracing System (CTS) database. The CTS records all movements at the level of the individual animal. Additionally, the import, export, birth and death of animals are recorded. The CTS was used to calculate the following variables:

1. Movements of animals that have spent any time in high incidence areas onto the herd. High incidence areas are defined as one and two year testing areas. The variable was calculated as the number of batches (a batch being identified as animals moving between the same pair of holdings in the same direction in the same day). The number of batches in the previous year was recorded.

2. On movements of animals that have spent any time in Ireland (Northern Ireland or the Republic of Ireland). This was recorded as binary based upon the previous year.

3. The total number of animals on each herd on the 1^st^ January of each year. This was calculated from the number of births, deaths and number of animals moving off and on during each year.

Data on the distribution of badgers were derived from the studies by Newton-Cross et al. [11] and White et al. [12]. Newton-Cross et al. [11] matched the most important habitat types for predicting main sett presence and absence with 1 km square habitat subclass data from the CEH Land Cover Map 2000, and calculated the probability of badger presence for each 1-km square. We then averaged the probability across all 100 1-km squares in a given 10-km square to obtain the average probability of main sett presence (per 1-km square) for each 10-km square in Britain, after White et al. [12].

Low incidence areas were defined as those parishes that were within four year testing areas for the entire period 2002 to 2008. This was because there is likely to have been a delay between the rise in incidence in four year testing parishes and their testing cycle being changed to a shorter period. Excluding parishes that changed status ensures that the “truly” low incidence parishes were identified and used for these analyses.

To evaluate whether there was a residual effect of restocking following culling during the 2001 FMD epidemic, herds that had animals culled as an Infected Premises, Dangerous Contact, Contiguous Premises or Slaughter on Suspicion herd were identified. These data were extracted from the Defra Disease Control System database.

### Statistical analysis

A multilevel logistic regression model was formulated in which the outcome (*Y*) is 1 if holding *i* in parish *j* recorded one or more confirmed incidents of bTB in year *k* and 0 if it did not. Thus, the model is fitted with holding nested within parish as a random effect and the year as a second random effect. Four different models were created depending upon the outcome:

1. All herds in GB.

2. All herds in one year testing parishes in year *k*.

3. All herds in two or three year testing parishes in year *k*.

4. All herds that were in four year testing parishes for the entire period 2002 to 2008.

An initial null model with no fixed effects was fitted. Candidate fixed effects were tested in univariate screening and those with p < 0.25 were included for testing in the multilevel model. Fixed effects that were significant at p < 0.05 were retained in the multivariable model. The effect on other predictors of the inclusion of a new predictor in the model was monitored and potential interactions investigated. The following candidate fixed effects were analysed:

1. The number of animals on the holding on January 1^st^ of each year derived from CTS. As this was a zero-inflated predictor it was categorised as 0 – 10, 10 – 100 and greater than 100.

2. The herd type according to VetNet herd table. This was reduced to five levels – beef, dairy, fattening, suckler and store. In this context the beef category refers to non-specialist beef producers whilst the fattening, suckler and store categories refer to producers who specialise in these aspects of production.

3. The x and y coordinates of the farm in meters, from the herd table in VetNet and transformed by dividing by 100,000. This was included to allow for any unmeasured spatial effects.

4. The number of batches of animals that the holding received from high incidence areas during the previous year from another holding in Great Britain. Taken from CTS and categorised as 0, 1–10 and greater than 10.

5. The total number of cattle in the 100 km^2^ grid cell that the farm is located in.

6. Whether the holding had any animals that were originally imported from Ireland (the Republic and Northern Ireland) during the previous year.

7. A history of bTB breakdowns – whether the holding recorded a breakdown between 1997 and 2001.

8. The predicted distribution of the badger population, based on Newton-Cross et al. [11] and White et al. [12].

9. Parish testing interval of the holding.

10. Whether the herd had animals culled during the 2001 FMD epidemic.

11. The country of the holding (England, Wales or Scotland).

In order to ensure that there was no spatial dependency in the residuals of the model, the variograms of the residuals of each year of the model were inspected to check for spatial dependency. Distances of up to 15 km were considered in the analysis.

Logistic regression analyses were carried out in the lme4 package [26] for the R statistical environment [27] and variogram analysis in the geoR package [28].

## Authors’ contributions

PRB carried out the analysis and drafted the manuscript, RO assisted with data processing and RRK conceived the study. PCLW and MRH supplied the badger probability data. All authors read and approved the manuscript.
